# Social determination of malaria in pregnancy in Colombia: a critical ethnographic study

**DOI:** 10.1186/s12936-023-04734-9

**Published:** 2023-10-06

**Authors:** Jaiberth Antonio Cardona-Arias, Luis Felipe Higuita-Gutiérrez, Jaime Carmona-Fonseca

**Affiliations:** 1 School of Microbiology, University of Antioquia, Medellín, Colombia; 2 School of Medicine, Universidad Cooperativa de Colombia, Medellín, Colombia; 3 School of Medicine, Universidad de Antioquia, Medellín, Colombia

**Keywords:** Malaria, Pregnancy, Gestation, Social determination of health, Qualitative research: ethnography

## Abstract

**Background:**

The meanings and experiences related to malaria in pregnancy (MiP) and its processes of social determination of health (PSDH) have not been reported in the world scientific literature. The objective was to understand the meanings and experiences of MiP, and to explain their PSDH in an endemic area from Colombia, 2022.

**Methods:**

Critical ethnography with 46 pregnant women and 31 healthcare workers. In-depth and semi-structured interviews, focus group discussions, participant and non-participant observations, and field diaries were applied. A phenomenological-hermeneutic analysis, saturation and triangulation was carried out. The methodological rigor criteria were reflexivity, credibility, auditability, and transferability.

**Results:**

At the singular level, participants indicated different problems in antenatal care and malaria control programmes, pregnant women were lacking knowledge about MiP, and malaria care was restricted to cases with high obstetric risk. Three additional levels that explain the PSDH of MiP were identified: (i) limitations of malaria control policies, and health-system, geographic, cultural and economic barriers by MiP diagnosis and treatment; (ii) problems of public health programmes and antenatal care; (iii) structural problems such as monetary poverty, scarcity of resources for public health and inefficiency in their use, lacking community commitment to preventive actions, and breach of institutional responsibilities of health promoter entity, municipalities and health services provider institutions.

**Conclusion:**

Initiatives for MiP control are concentrated at the singular level, PDSH identified in this research show the need to broaden the field of action, increase health resources, and improve public health programmes and antenatal care. It is also necessary to impact the reciprocal relationships of MiP with economic and cultural dimensions, although these aspects are increasingly diminished with the predominance and naturalization of neoliberal logic in health.

## Background

The process of social determination of health (PSDH) of Latin American Social Medicine addresses health-disease problems from a processual perspective (holistic, historical, dynamic, multilevel and multidimensional) in which health outcomes are analysed in the context of the economic relations of accumulation, property and power. PSDH is aligned with critical theory proposing that the people reflect the social history of their group and social processes determine individual behaviour because the social dimension enables and conditions personal interactions, political events, and economic relations. Therefore, social processes also determine the worldviews, meanings, and experiences of the subjects [1–3].

For critical health theory, social relations within capitalism are important to explain health outcomes; the dialectic relations of singular, particular, and general levels are the *core* of the methodological design, and the centrality of the subject reveals the importance of transcending the disease dimension of the biomedicine-positivist approach toward subjective and intersubjective domains (illness and sickness dimensions of medical anthropology) [1–3]. Despite the importance of critical public health approaches, they have not been applied in scientific publications on the social determinants of malaria [4]; in malaria in pregnancy (MiP) the investigative hegemony has been the biomedical-positivist model, with a marginal investigation of PSDH in some qualitative studies [5–7].

Qualitative studies describe, explain, and understand popular concepts, and they privilege the subjective and intersubjective dimension of social reality [8, 9]. Qualitative Health Research (QHR) addresses meanings, experiences and behaviour related to resistance or adherence to healthcare workers (HWs) recommendations, social representations of health, community responses to healthcare topics, social determinants of disease, and explanatory models of health-disease process from the popular perspective [10, 11]. A recent scoping review showed that QHRs are used for health policies and systems in the following axes: policy or programme content, environment or implementation, and structure-function of health systems [12].

Despite the advances in QHR, critical approaches are scarce, and phenomenological-hermeneutic studies predominate [1]. Regarding MiP there are no studies about PSDH and most QHR focus on content (frequency of concepts or words to infer meanings) or thematic (focused in pre-established categories) analysis about acceptability barriers of insecticide-treated bed nets, intermittent preventive treatment or case management [6, 7]. There is no social study of MiP in Colombia or America, that allows an initial identification of their PSDH.

Additionally, some authors propose mixed methods to study PSDH; however, mixed studies in this topic present several weaknesses that could be overcome by focusing on QHR, particularly critical ethnography:

(i) Mixed designs make greater emphasis on measuring inequalities in health outcomes, and qualitative components are used to explain the gaps and identify generating mechanisms of inequity, sacrificing the depth and breadth of the qualitative findings;
(ii) It would not be determined whether in malaria—endemic areas reproduced the neoliberal *status quo* in health, characterized by privileging insurance over care or prevention, targeting on biomedical recommendations that privilege individual responsibility over structural causes of MiP, and ignoring cultural or popular perspectives of the disease;
(iii) The way of understanding and dealing with MiP among those affected, their explanatory models of the disease and PSDH would not be known in depth; besides, the explanatory contexts of the meanings, behaviour and experiences related to MiP would not be addressed;
(iv) The importance of subjectivity and intersubjectivity in the social construction of MiP would not be revealed, or other concepts, such as illness, sickness, health, healing, and wholeness, that could guide changes in the control programmes and policies.

Therefore, this research was carried out, which is the first in Colombia that aims to understand the meanings and experiences of MiP and explain their PSDH from the perspective of those affected in an endemic area, 2022.

## Methods

### Place of study and context

**Fig. 1:**
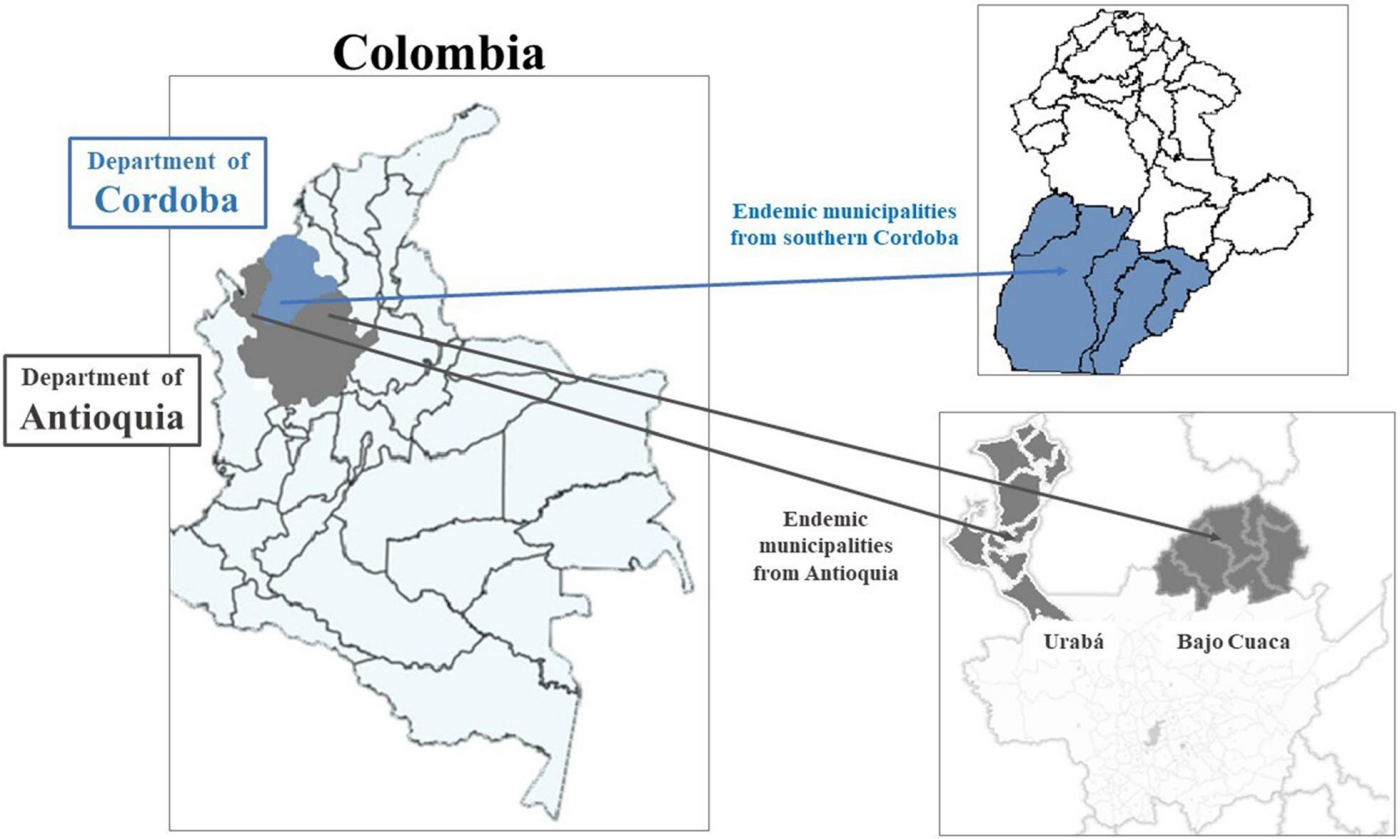
The site of study

The study was conducted in northwestern Colombia, in the departments of Antioquia (subregions of Bajo Cauca and Urabá) and the Southern Córdoba (Fig. 1). This region reports the highest number of malaria cases in the country, it has a high and constant transmission, with circulation of *Plasmodium vivax* (60–70%) and *Plasmodium falciparum* (30–40%) [13].

The subregions (Urabá, Bajo Cauca, Southern Córdoba) have similar living conditions, characterized by predomination of dispersed rural territory, a high proportion of unsatisfied basic needs, monetary poverty, precarious material living conditions, informal employment, few people with their own homes or land, concentration of wealth, mining (mainly artisanal or illegal) and livestock as the main economic activities, land use in agricultural activities, armed conflict, and low presence of welfare state programmes [14–17].

**Fig. 2:**
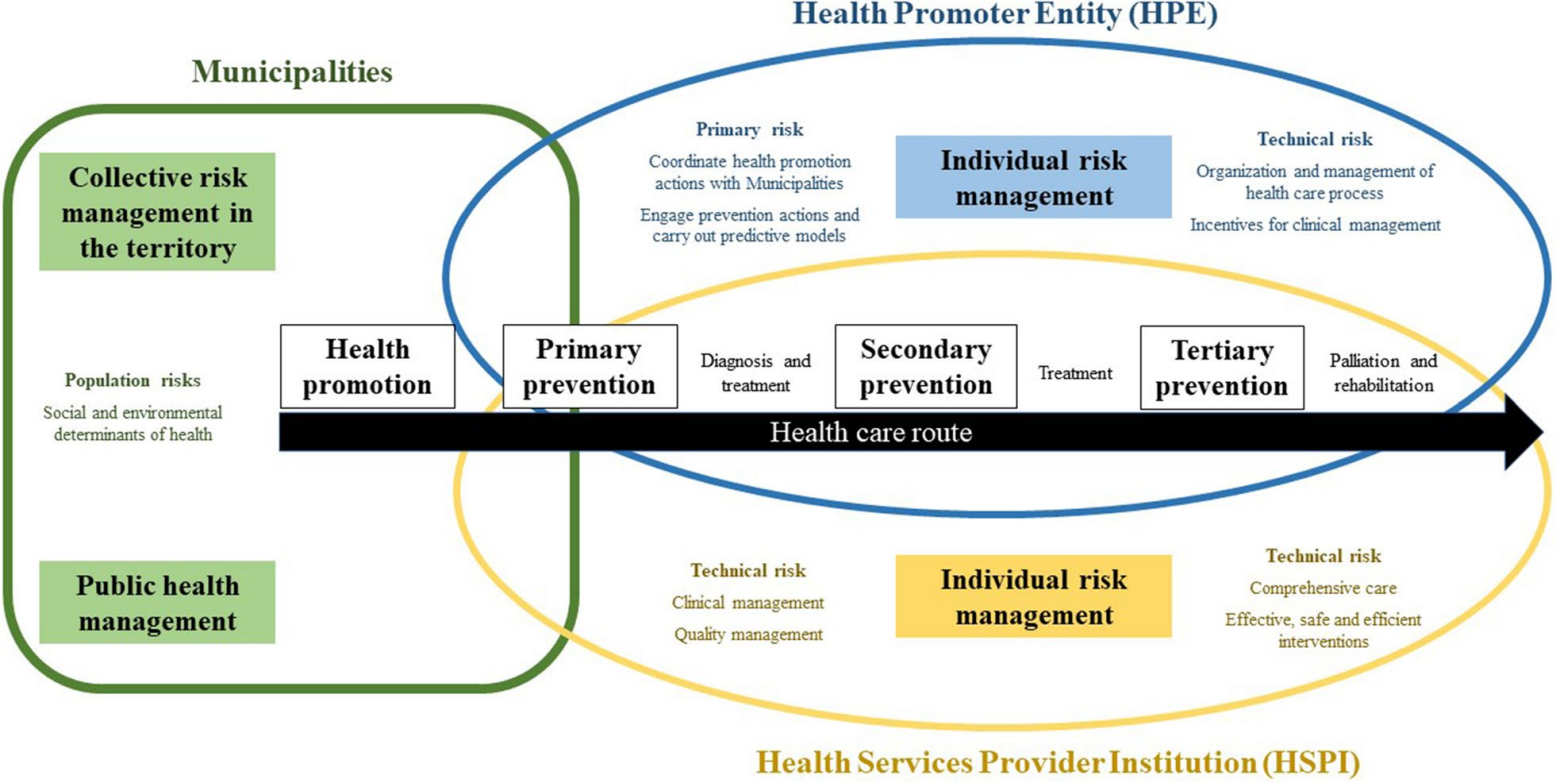
Actors of the health system in Colombia related to malaria care

On the other hand, it is important to explain some responsibilities of the actors of the Colombian health system to provide prevention, diagnosis, and treatment of MiP (Fig. 2). The Ministry of Health, through the administrator of the resources of the general system of social security in health, transfers the money to the Health Promoter Entity (HPE) and to the municipalities to pay for the healthcare. The HPE is responsible for the affiliation of people to the social security in health and organizing and guaranteeing the provision of health services by payment to Health Services Provider Institutions (HSPI) (hospitals, laboratories and clinics that provide the care service to patients).

### Type of study

Particularistic critical ethnography (study of a specific human group) was applied, it is ideal for studying vulnerable populations because it seeks to understand the dominant discourses (the “correct” way of thinking, speaking or representing a situation) and to recommend ways to correct them when they exacerbate social power inequalities [18–20]. Critical ethnography is an interpretation of the culture under study, charged by the vision of the ethnographer as one more participant during the investigative process, who reflects the positions and values of the participants favouring reflexivity [19, 20].

Critical ethnography addresses the culture of social groups from their diversity, conflict, and cohesion, keeping in mind certain cultural relativity (groups are different, despite having something that makes them equal in some domains) and that culture is positioned unequally in power relations. It highlights the importance of critiquing (and from there transforming) social, political, cultural, economic, ethnic, and gender structures that are unfair because they contradict human rights and social justice [21–23].

### Participants and sampling strategy

Through an informal conversation with HWs, municipalities with an ongoing armed conflict were excluded for field work. In these types of municipalities, two hospital managers gave an in-depth interview outside their locality. In the remaining municipalities, hospital managers and health secretaries were invited by written communication, obtaining a positive response in six (two from Córdoba, three from Bajo Cauca and one from Urabá).

The number of participants was determined with theoretical saturation for the PSDH, *a priori* thematic saturation for pre-established categories, and inductive thematic saturation for emerging categories. Saturation was defined when the new participants repeated previous information [24]. With these process were included three microscopists, two community health promoters, six bacteriologists, eight nurses (three from promotion programme, three from epidemiological surveillance, one from emergency department, one from chief staff), five physicians (two service coordinators, one from emergencies and two from antenatal care—ANC), two local hospital managers (in addition to the two previously indicated), two municipal health secretaries, and a nurse coordinator of the vector-borne diseases departmental health programme.

For pregnant women, a selection process by the maximum variation was applied to represent a broad population spectrum; in this way, quotas were defined by health affiliation regime (subsidized, contributory and without affiliation); by age group (under 20 years, between 20 and 24 years, and between 25 and 36 years); by education (without education, with primary or secondary education), and by origin (urban or rural). In each quota, the number was defined by saturation; in total, 46 pregnant women were included.

#### Reflexivity and characteristics of researchers

All researchers have training in epidemiology and parasitology; they have conducted research on MiP in the study region. JACA and LFHG have more than 10 years of experience in QHR and postgraduate qualifications in social sciences. JCF has more than 30 years of experience in malaria research and has carried out research on MiP since 1995. JACA designed the collection instruments, and other researchers validated them.

JACA underwent immersion in the study area in the company of a microscopist and nurse recognized and accepted by the community. JACA filled out a reflective field diary (taking one’s own experience in the field as an object of reflection) to describe opinions, reflection on his thoughts and observations in the field, and reactions of the participants to his presence. The researcher did not know the study participants and it was defined *a priori* that no clinical or educational activity would be carried out before the fieldwork, to capture daily issues or the natural environment of ANC.

JACA and LFHG carried out the coding and categorizing the videos and photographs of the diagnostic post, the hospital and some villages, and the transcribed interviews. All the researchers were involved in setting the codes and categories for the analysis. Three researcher guaranteed reflexivity during the analysis and writing, contrasting their interpretations and predominant theoretical currents (PSDH, rurality, gender and health; medical anthropology, Colombian social security health system, malaria control and epidemiological surveillance of vector-borne diseases).

#### Ethical issues

The declaration of Helsinki and Resolution 8430 from Colombia were applied. The study was classified as minimal risk and was endorsed by the Ethics Committee of the SIU (*Sede de Investigación Universitaria* in Spanish) Act 21-101-961. The participants signed the informed consent (of legal age) or assent (under 18 years of age), obtained in writing, it was also signed by a witness external to the research group, and a HW who explained its content. The consent process informed participants about the objectives and justification of the research, the risks and benefits of participating, the mechanisms to guarantee confidentiality, the importance of the willfulness and rights, the possibility of withdrawing from the study at any time, and the endorsement to publish the results as anonymous information.

#### Instruments and data collection

Data collection and analysis were carried out between January 2022 and March 2023, triangulating the information generated with the following instruments. Semi-structured interviews for the pregnant women with three questions (based on their answers, a conversation was generated to capture the main meanings and experiences related to MiP): (i) tell me how your pregnancy process has been, (ii) tell me how you came to the ANC programme, what have been your most important experiences in this programme, and (iii) tell me about malaria (what do you know, causes, transmission, prevention and treatment).

In-depth interview was applied to hospital managers based on a question: could you describe the structure and operation of the ANC and MiP control programmes in your hospital? Based on their response, a fluid conversation about emerging aspects was established. For HWs a semi-structured interview was applied with the following items: (i) description of the ANC and malaria control programmes, (ii) key factors for the prevention, diagnosis, treatment and surveillance of MiP, and (iii) significant experiences with the care of pregnant women and MiP control.

A focus group discussion with pregnant women and another with HWs explored about knowledge, attitudes, and behaviour related to MiP and its PSDH. The participant observation was made during the interviews and focus groups, highlighting the following aspects: interactions of participants (dominant, passive), risk of censorship in some topics, positions of conformity with the answers from other participants, psychological (incl. shyness, fear, age) or social (incl. occupation, rurality, schooling) factors which may affect the answers. Observation was carried out at the malaria diagnostic post, in hospital waiting rooms and ANC to analyse the interaction between pregnant women, HWs, communication mechanisms in healthcare, waiting times, information about care routes. Non-participant observation was also made in the villages with highest endemicity to analyse barriers to access to healthcare, environmental risk factors, application of community control measures, vector management, material living conditions, typologies of housing, roads-facilities of access. These observations were recorded with videos, photographs, and a field diary taken by JACA.

The field diary was filled out with various types of notes: (i) reflective (previously described), (ii) methodological to improve data collection, making language adjustments, analyses of discursive and non-discursive forms of the participants, (iii) theoretical about conceptual frameworks relevant for constant-iterative analysis, and (iv) behaviour of the participants and their environment (etic perspective).

#### Data processing and analysis

Data were transcribed and anonymized by assigning an initial letter by source (I interview, D diary, O observation, G focus group), a second by a participant (G Pregnant woman, HW healthcare worker), and a sequential number. Transcriptions were read for phenomenological-hermeneutical analysis identifying significant texts to start the coding process with pre-established and emerging codes. Similar codes were grouped to establish categories, subcategories, properties (attributes that delimit the content of each category), and dimensions (variation range of a property). Categories were related to four levels: structural problems of the study region, public health and ANC programmes, malaria control programmes, and meanings-experiences of singular level. This process made it possible to generate a matrix of processes that explains most of the cases, situations, meanings, experiences, and PSDH of MiP found in this cultural group. The results were shared with the participants to receive their endorsement and feedback, discussing whether this configuration constituted a reasonable explanation of what is happening in the region.

#### Techniques to enhance trustworthiness

Processual and critical ethnography were applied, guaranteeing the application of the characteristics of holism (trying to capture the variations of the categories), contextualization (with a dense description of the study region), reflexivity, the articulation of the emic (describes the facts from the perspective of its agents) and etic (describes the facts from the observer’s viewpoint) perspectives, saturation of categories, and triangulation of methods, researchers, and theories. Credibility criteria were followed through prolonged contact with the participants, auditability of the investigators’ interpretations and validation of the results with the participants, and transferability or applicability to other endemic areas.

## Results

**Fig. 3:**
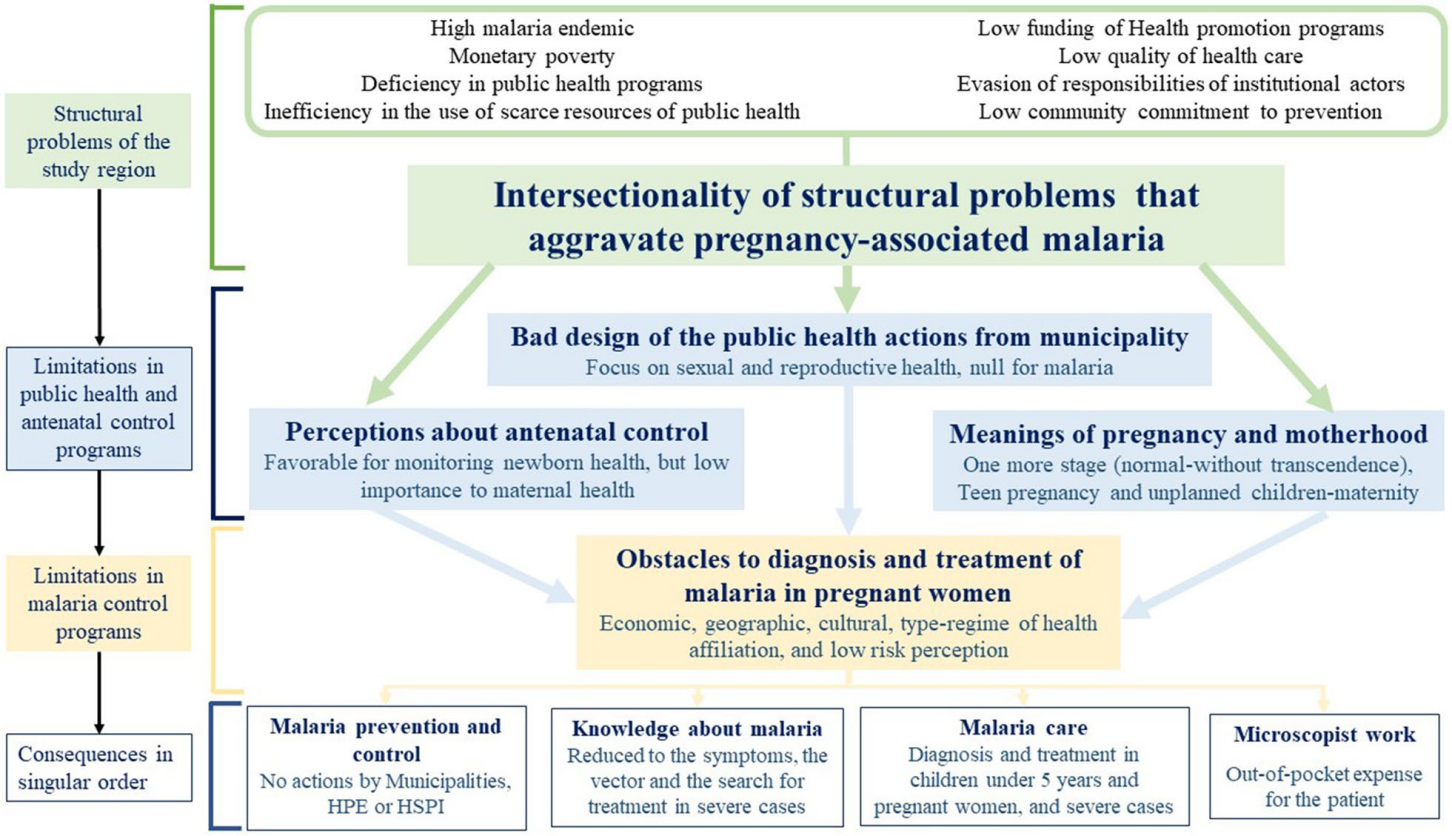
Categorical relationships on the structural, institutional, and individual processes that prevent optimal control of malaria during pregnancy

Figure 3 shows the categories of the study, their grouping, and determination relationships (structural processes determine the findings of the next hierarchical level). Processual relationships of four levels were identified: (i) structural problems of region studied that converge with MiP, (ii) limitations of public health and ANC programmes, (iii) limitations in malaria control with obstacles for MiP diagnosis and treatment, and (iv) consequences of previous levels on the singular level, where the limitations identified were for prevention and control actions, lacking knowledge about MiP, care of MiP restricted to cases with high gynecological-obstetric risk, and the importance of the microscopist.

### Intersectionality of structural problems in the endemic region studied that aggravate the epidemiological profile of malaria in pregnancy

Socioeconomic, political, cultural, and environmental problems converge in the area studied, participants mentioned eight: high endemic malaria in rural areas, poverty, deficiency in public health, inefficiency and corruption in the use of public health resources, scarce financing of health promotion programmes, low-quality healthcare, evasion of responsibilities of institutional actors, and absence of community commitment with malaria prevention.

Participants know a high number of malaria cases in rural areas from their municipalities; despite this, they refer to MiP as an event without importance in the municipality, the community, or at home. This naturalization of malaria endemicity leads to an indifference toward prevention and care; the actions are reduced to the diagnosis and treatment of symptomatic cases not resolved with natural or pharmacological self-medication.

The second structural problem was monetary poverty due to low income, deficiencies in the material conditions of housing (mainly in rural areas), problems accessing good jobs (the majority is agricultural work hired by the day), and economic difficulties in accessing a good nutrition. The participants refer to various feedback mechanisms for poverty and malaria: (i) poverty prevents having a good state of health due to not having adequate food and difficulties in accessing health services; (ii) poverty is more serious in the most remote areas, which makes access to hospitals difficult; and (iii) disease generates physical disabilities that do not allow people to work, which increases monetary poverty. Some testimonials expand the content of this category:

*“Our dispersed rural population is quite large, more than 60%. We have many villages in dispersed and remote areas, to six or eight hours from the urban area. We have communities that to get to the hospital, must walk or ride a horse, another journey by water or motorbike, another by bus or car. We have quite dispersed communities that are three or four hours away by a vehicle, such as a motorcycle. This makes it difficult for many of our cases of malaria in dispersed rural areas to reach the urban areas and hospitals. And taking into account the economic factor, also the low economic affordability that many people must travel to the hospital, so that impacts their poor health”* (I.HW.2).

*“For the baby’s exams, the urgent ones, I had to pay privately* (mainly ultrasounds). *Until now I haven’t taken all the exams because I haven’t had money to go to the hospital. So, since I do not have money, everything is difficult for me in my health, in my appointments, and in those of the baby. I do not have a job either, so I do not have money, and without money I can’t get to the hospital”* (I.P.4).

The third category refers to shortcomings in public health programmes: (i) having a low budget for the needs of the municipalities, (ii) administered (coopted) by the Mayors (which encourages corruption of political groups), (iii) poorly designed because they are restricted to the regulatory requirements without incorporating the specificities of each municipality, (iv) not having methodologies to prioritize health problems, (v) having low population coverage, and (vi) having a reductionist vision of public health (health education is reduced to talks or flyers, community management is restricted to sporadic brigades in a few villages, epidemiological surveillance is practically nil).

*“The social development secretariat does not have a presence in the communities. We have few activities; we do not move much to the villages because people do not arrive and due to geographical difficulties. On malaria, the public health part has been handled with education, talks are scheduled, flyers and folded leaflets are distributed, but the community is indifferent”* (I.HW.4).

*“It is very difficult for us to do an active search due to the size of the hospital, and the low number of personnel for the demand for health services that the municipality has”* (I.HW.1).

Additionally, the inefficiency in the use of scarce public health resources was highlighted, Mayors award contracts for public health projects to their friends. There is also corruption in the management of public resources and lack of financing for health promotion programmes because the system is oriented to the care of the disease.

*“There are shortcomings in budget allocations. I consider that the guidelines are fine, but there is a very delicate issue and that is corruption. If in the municipality we managed better the resources we have, we would have better results. But we spend them badly, for example, they give us 100 pesos for health, and we really spend 30. On other occasions, we must work on the issues that the provider indicates, and not in those that are most important for the municipality”* (I.HW.6).

Another structural problem is lack of quality in healthcare, attributable to the low response capacity of local hospitals, negative attitudes in care provided by HWs, test for MiP diagnosis that are not applied at least every trimester of pregnancy, absence of epidemiological surveillance with active search for cases (the system is aimed at passive search, only the cases of symptomatic patients who consult in the hospital are registered), excessive paperwork in authorization of exams, multiple displacements to other municipalities that have a higher level of complexity of attention, among others that are illustrated in the following testimonies:

*“Many pregnant women arrive at ANC without ultrasound; They do not have psychological or gynecology care. It is not known if the problem is from the HPE that does not authorize the exams, from the HSPI that has delayed the schedule, or from the patient who did not bring what she needed to receive care”* (I.HW.11).

*“I feel that the bacteriologist does not make a good diagnosis of malaria in the urban areas, and we have practically nothing for rural areas. Until about five years ago, a malaria test for maternal mothers was not required. Today, it is requested, but not all of them do it because they forget because they do not know how to do it from the hospital because they do not have the money or because many rural doctors [*recently graduated physicians*] come to this hospital and they do not do the screening for gestational malaria”* (I.HW.3).

Participants also referred to the evasion of responsibilities among the actors of the malaria control programmes. HSPI, HPE and health secretariat must have malaria programmes, but these actors postpone their responsibilities assuming that the others will take up these responsibilities; none assume their functions, as corroborated in the following testimony:

*“There we have a great shortcoming, the whole issue of public health, even though it is in norms, the issue of malaria is not met. Fortunately, we have very committed microscopists, and they make the thick blood smears for the maternal ones. But here there is no clarity about who should be responsible for taking samples for malaria and search for cases: should the municipality, the Department, the Hospital, the HPE do it, finally whose obligation is it?”* (I.HW.9).

Regarding collective and community actions in favour of health and malaria control, the pregnant women reported that they do not know about these issues, they do not participate, and there are no leaders for these initiatives in their villages, evidencing a lack of community commitment with the prevention of MiP because for the community it is more urgent to attend to other situations such as their low income, insufficient food, geographic and economic barriers to guarantee the right to health, among other problems related to poverty; while health workers do not encourage their participation in malaria control actions.

*“People kind of get used to it: do you have intermittent fever? That is malaria. This is something normal for the community, they know that it can give them, that they can repeat it. In that normality, they got used to not paying attention to it, they got used to that disease and that is why it stopped being important for the community. For rural areas, if they are not told about malaria from the hospital or the health department, people will not think about it”* (I.HW.2).

### Limitations of public health programmes in municipalities and antenatal care programmes in hospitals

At this level, three categories were identified: poor design of public health programmes for pregnant women (focused in mental, sexual, and reproductive health), perceptions about ANC (mainly benefits for good development of the child), and meaning of motherhood.

The participants cited the following shortcomings in the design and operation of public health programmes in their municipalities: (i) confining public health responsibilities to local mayors’ offices has resulted in them not using economic resources efficiently and transparently, (ii) every year public health resources are spent on the same activities (flyers, talks, screening of some events with the same contractors), (iii) by national guideline, investments in mental health (mainly depression or suicidal ideation) and sexual and reproductive health (talks on planning and screening of sexually transmitted infections) must be demonstrated, without evaluation of their impacts, and (iv) absence of actions to control malaria; if the health authority requests management indicators for malaria control, officials report data related to vector control that is carried out with budgets assigned to dengue control.

*“The Ministry of Social Development is an entity, a dependency that is in charge of education, health, sports, culture, and vulnerable population. It is a large number of things for a single official”… “On vulnerable populations, these are the populations: indigenous people, displaced population, the elderly, and abandoned children. Everything must be done with low money”… “Basically in the municipal committees the medical part is worked on, public health is sought, especially when we have outbreaks or peaks of diseases, mainly dengue”* (I.HW.26).

There is no specific budget for MiP control, which has led to several serious situations: malaria control actions are carried out based on passive and paternalistic perspectives; while the administrators of economic resources of the malaria programme have a great dependence of initiatives external to the ministries of health such as projects of non-governmental organizations, Red Cross, international collaboration or universities. Besides, this situation increases out-of-pocket expenses for people who require timely diagnosis and treatment, and lack of action in priority groups with higher clinical and epidemiological risks.

*“We work few malaria activities, and it is malaria in general with some flyers or brigades; We do not prioritize any group as pregnant women. These groups, such as pregnant women or children, have them as a priority population for hospital care when they present serious symptoms”* (I.HW.16).

ANC is valued for allowing us to know the state of health of the baby, with scarce reference to benefits for mother’s health. Only pregnant women affiliated to the contributory regime are screened for MiP in ANC checkups; while all those affiliated to the subsidized regime indicate the absence of thick blood smear, this test is performed only on pregnant women who pay for it as an out-of-pocket expense.

*“ANC seems fine to me. I say good because there they are awaiting the baby. The most important part of the control are all the tests that are sent to one to see that the baby is doing well. The control is so that everything goes very well with the baby and it is born well”* (I.P.12).

In the meaning of maternity, it is indicated that this is one more stage in a woman’s life, it constitutes normal fact that is expected in being a woman. Most of the interviewees speak of this stage as an exclusive responsibility for women since men are absent or are limited to the material provision of the home. In few cases, this stage is referred to as something planned and desired (mainly in pregnant women in urban areas and with high levels of education); there is a high frequency of adolescent mothers and unplanned pregnancies.

### Limitations of malaria control programmes for pregnant women

This level describes some economic and geographical obstacles; cultural issues; social health security, and risk perception explain the absence of diagnosis for asymptomatic pregnant women, MiP underreporting, among other problems related to surveillance, prevention, and control of MiP.

Economic and geographical obstacles:

*“Here most of the territory is dispersed rural. Many pregnant women live in areas where the hospital is not present. Therefore, when the pregnant woman has intermittent fever, they say that this is malaria, they self-medicate with chloroquine because they do not have money to go to the hospital, and it is another case that we do not detect”* (I.HW.3).

*“We have pregnant women in some villages that are far from the urban areas, it will be very difficult for these pregnant women to attend ANC, either due to economic or geographical factors; They must travel by horse, buy a motorcycle, car or boat, to be able to reach their health post, and these costs are very high, so they are constrained and it is better not to travel to the hospital”* (I.HW.1).

Cultural differences or cultural diversity that is manifested in healthcare:

*“We have pregnant women who do not consult ANC, and We only see them at delivery. Here, there are many indigenous reservations and one of the biggest problems in ANC is the indigenous population, due to their cultural issue they do not attend ANC check-ups and their deliveries are in their communities with midwives. And we in the hospital do not have direct contact with them”* (I.HW.24).

*“The issue with indigenous communities is that their pregnant women generally arrive at the hospital with the gestational product, just because they seek to legalize the entire issue of the child’s civil registry because it is required by Colombian law but not because they want to receive our attention”* (I.HW.30).

Healthcare barriers for pregnant women also included an affiliation to different HPE, pregnant women who belong to HPE whose geographical coverage is in a different municipality, HSPI problems to guarantee that the HPE pay for the services they provided to their pregnant women, and the absence of hiring personnel with training in MiP diagnosis.

*“There is a serious problem for the maternity program and it is the issue of mobility, today they are in one municipality and tomorrow in another municipality, and they do not transfer their HPE. At the hospital, we cannot deny care, but we know that no one will pay us for this care and that could affect the hospital’s finances. We should talk about the financial issue with representatives of the Government, the HPE and HSPI to solve this issue”* (I.HW.8).

Finally, to this category are added different narratives that reflect a lacking perception of the risk of becoming infected and ill with MiP:

*“Malaria is serious but not always. The truth is that this is hardly talked about here. At ANC check-ups they have not talked to me about malaria, nor at school, I don’t know anything about malaria but I have never gotten sick from it”* (I.P.21).

*“In my environment, in my house, in my work, there is nothing that endangers my life or my health. Generally, here, everything is very healthy. I hardly listen to things about malaria; but I don’t think that I am affected by this disease, and if I get a fever, I know what drug to take”* (I.P.3).

### Consequences at the individual level

All of the themes reported in the above sections have consequences at the individual level for pregnant women, in dimensions of MiP problems not adequately captured in biomedical research and not included in health planning that is generally carried out remotely from the lifeworlds of these pregnant women. These consequences are grouped into three axes: (i) absence of MiP diagnostic, although the guidelines indicates that ANC must include the application of at least one thick blood smear each trimester; pregnant women indicate that they are only performed when they are symptomatic or they pay it as out-of-pocket expenditure on health, (ii) MiP care restricted to cases of high obstetric risk, (iii) lacking knowledge about risk factors and consequences of MiP.

*“Who told you that the mosquito gives malaria, if the mosquito gave malaria, everyone in my village would have it. Malaria occurs because one gets wet with rainwater, or because one bathes in a stream, or is weak”* (I.P.11).

*“Generally, I don’t know anything about malaria, nor have they told me in the ANC check-up that I should do something for malaria. I also know that if I get a fever and a lot of pain and chills, I come here to the post and pay the ten thousand for the thick blood smear, and if it comes out positive, I go to the hospital pharmacy. For the rest, I don’t hear anything about malaria in this town”* (I.P.13).

Regarding the microscopist job, it is important to indicate that these people are nursing assistants and health promoters who received training for the diagnosis of malaria, certified by SENA (*Servicio Nacional de Aprendizaje*, in Spanish). None of the interviewed group had a formal employment contract. This situation, in the voice of the hospital managers, is because malaria has no budget and, therefore, microscopists cannot be hired. The last contract they remembered was more than 8 years ago with the government, but later, this responsibility was delegated to each territorial entity (municipality or HSPI). Despite this lack of hiring, the people of the community continue to have microscopists as the reference personnel for the diagnosis of malaria, who are paid ten thousand pesos for each thick blood smear.

## Discussion

This multi-level, big-picture approach is in many ways a strength of the manuscript because argues the intersectional nature of the PSDH, highlights the complexity of the problem, and makes a strong case for new, socially-informed approaches to addressing MiP with critical theories. However, including such a large number of themes in one paper may also be a limitation because necessarily reduces the depth of discussion of each PSDH; which should be corrected in subsequent studies. This research focuses on the breadth and interconnected social dynamics of MiP, because critical theory in health demands a greater study of meso (particular-community) and macro-social (incl. political, economic) processes, to achieve the transformation of the harmful and unfair realities previously described in the results.

At the individual level, participants highlighted the absence of prevention and control actions in their home and village, lacking knowledge about MiP, hospital consultation in symptomatic cases and with risk of complications, and out-of-pocket expenditure on health to receive a diagnosis. These findings coincide with the synthesis of malaria QHR from Colombia, in which there was high knowledge on mosquito, fever, and weakness, but poor on other topics such as risk groups, surveillance, and prevention. The results also converge in the fact that community actions in health to prevent and control malaria are not reported, and that biomedicine is consulted only in advanced symptomatic cases, with exception of a low number of people who have had previous malaria or perceive high risk of contracting it [25].

This situation has also been described in the qualitative component of mixed studies on MiP, and a systematic review of QHR in MiP that reported lacking knowledge about MiP and its consequences, lacking perception of the risk of becoming infected, and poor knowledge about prevention [6, 7]. This demonstrates multiple challenges for health education, information and communication programmes, since the individual cognitive dimension in this population is determined by social, cultural and economic processes, which are excluded in this type of health strategy, despite having a greater explanatory power on individual behaviour.

Specific findings of the type of Colombian health system were highlighted, referring to the out-of-pocket expenses associated with thick blood smear, which has been described as an important economic determinant in other populations. Although this was narrated by the participants as a singular level event, with the absence of public health community actions by the institutional actors, these aspects will be discussed at the next level that addresses social security issues in health [26–28].

Singular level findings were determined by the economic, geographic, cultural and health system barriers to access malaria control programmes, in interaction with a lacking perception of the risk of becoming ill and dying for this disease. These events have been recurrent in the QHR on MiP, and in other countries they have been used to explain less effectiveness of control strategies (such as intermittent preventive treatment, use of nets, detection and treatment of cases) [7, 25]. These barriers coexist and would explain the high frequency of asymptomatic MiP, which increases the risk of severe malaria, placental malaria, congenital malaria, anaemia, and low birth weight, among other outcomes [29–31]. These recurring findings in various studies call for urgent actions on contextual barriers, to improve access to different health services for MiP, especially those related to timely diagnosis that improve maternal, congenital, and neonatal health.

At the third level of PSDH, the participants located the poor design of health programmes aimed at pregnant women (focused on mental, sexual and reproductive health), without specific budget for MiP, and characterized by shortages, inefficiency, and without considering the specificity of each context. This can be explained by the low proportion of health system resources directed to public health; some trends show 90% of resources allocated in the provision of healthcare services and 10% in public health actions [32].

This is serious considering the high costs associated with pregnancy in Colombia, which require efficient use of health resources because they represent around 3.3 trillion pesos for households; 63 billion for Colombian companies (mainly for maternity leave); 5.7 billion to the State and 3.3 trillion to the Health System. Calculations for each pregnant woman indicate that her care cost 6.8 million, which can rise to 390 million when she dies prematurely [33]. In the subsidized health regime, the cost of care for each pregnant woman was 4.4 million (expected cost of 2.5 million), reporting greater consumption of resources in pregnant women who did not receive the minimum activities of their ANC in the first level of care complexity. This level only fulfilled 52% of the activities that must be carried out. Costs are 80% higher than budgeted, mainly due to charges for hospital stays, medicines, and caesarean sections [34].

The foregoing interacts with socio-anthropological issues linked to the low value of pregnant woman (life of the child comes first) and meanings of motherhood that assume this period as a normal condition for women; this finding differs from the few reports on this topic in Colombian pregnant women. The study of the Gómez found that the main representations of pregnancy are the postponement of studies, family and economic dependency, and the low importance given to the baby among primiparous women; while in multi-pregnant women, they are associated with school dropout, family and economic independence, and the baby as the center of representations of love [35].

Although explicit categories on the gender approach did not emerge in this research, it is necessary that further studies investigate power relations according to gender, the degrees of autonomy of pregnant women in decision-making related to their health, and other issues related to the gender approach, given that previous QHR have indicated its importance for understanding some sociocultural determinants of MiP [7, 25].

At the general level, it was reported the intersectionality of MiP with structural PSDH such as the naturalization of exposure to malaria, poverty and its feedback with the disease; low budget, inefficiency and corruption in the use of resources for public health; lacking quality of maternal care, network of institutional actors that evade their responsibilities, and lack of community commitment to actions to control MiP. This level is more important for long-term actions, for intersectoral, interdisciplinary, or intersectional policies for the prevention, diagnosis and treatment of MiP, from a critical public health approach.

The naturalization of exposure to malaria could be understood as the presence in several generations of multiple risks not mitigated, socially reproducing a kind of resignation to face the disease. Subsequent studies should deepen the understanding of the mechanisms that guarantee the social reproduction of these positions against MiP, and return to sociological research that studies the processes of legitimization and maintenance of social order. This naturalization of exposure or living with the disease is also an indirect way of demonstrating that the efforts of more than half a century of malaria interventions, since the creation of the National Malaria Service and the Eradication Campaign, have not resulted in the expected results. Quantitative studies have shown that during the short-term eradication period (1959–1969) a decrease in cases was achieved, but in the period of intensification of control for eradication (1970–1979), there was an increase in the number of cases, due to financial problems that prevented the maintenance of the downward trend [36].

The null community commitment with MiP control was perceived by participants as problematic, which has been described in others QHR on malaria in Colombia, as one of the main challenges and contributions of social studies of the disease [25]. Communities generally want to be in good health, but rarely get a say in the healthcare interventions that are provided in them; they have not been informed, educated or given a chance to choose the kind of healthcare that they need. Additionally, the participants in this study identified the link between MiP and poverty, and described various feedback pathways, which coincides with previous studies that have delved into the study of this relationship and have concluded that malaria control also requires actions against monetary poverty [37, 38].

Low budget and inefficiency-corruption in the use of resources for public health were also identified; this constitutes a great challenge given that eradication programmes of 1950 failed for financial reasons [36]. In the current structure of the Colombian health system, there is no effective control against corruption. In interviews with some experts, at least 40 modalities have been identified with which HPE, HSPI, doctors, laboratories and patients steal money from the sector [39]. Some investigations have pointed out that inefficiency and corruption, lacking quality of care, and a network of institutional actors that evade their responsibilities, are a consequence of the structure of the insurance system that fragments the functions, makes more complex financial relationships, complicates the monitoring of the use of money, prevents open or public billing systems, it does not provide sufficient economic and legal resources for control agencies, and it does not allow the training of a sufficient number of health professionals, among other structural problems [40].

The different levels of PSDH in MiP show the difficulty to achieve an effective control of the disease, among other reasons, because the care of MiP in Colombia reproduces the intrinsic limitations of the following models: (i) aetiopathogenic or microbial conception as support of malaria control, (ii) epidemiological surveillance based on individualistic linear relationships of variables, (iii) public health with hegemony of biomedical approach focused on disease (exclude illness and sickness dimensions), (iv) social security based on economic rationales of liberalism and utilitarianism, (v) conception of health that does not reveal the power relations among the actors of the system (Ministry of Health, Secretaries of Health, Mayors and Governors, HPE, HSPI, HWs, researchers, patients, communities). This structure is useful for the reproduction of an unfair socio-sanitary order for pregnant women exposed to MiP.

The main limitation of this research was the impossibility of having access to some municipalities due to the armed conflict. It is also important to indicate that the analysis was based on the multilevel dialectic (general, particular and singular) of PSDH, without delving into other relevant constructs such as the banalization of MiP reflected in the low financing of control programmes, prioritization of other events of public health, lacking the quality of maternal and fetal healthcare, lacking the perception of the risk of becoming infected and the absence of social mobilization in favour of the health of pregnant women. These underlying phenomena demonstrate the power of neoliberal rationality and its reproduction in all actors of the system health. This rationality corresponds to a less coercive but more intense exercise of power because it generates hardly perceptible processes that reproduce individualistic and utilitarian economic logics, through various devices applicable to this research, such as expertocracy (experts focused attention of pregnant women on mental or sexual health), psychologization and individualization (structural factors are not intervened, it is assumed that prevention and attention of MiP is an individual responsibility), the reduction of social spheres (social practices are not recognized and MiP control is restricted to costs and benefits), and production-consumption of individual freedom (the pregnant woman as her own “entrepreneur”, her poverty and MiP is the consequence of personal decisions without taking into account her material living conditions) [41].

The strengths of this critical ethnography included revealing various levels in the PSDH in MiP in the study region, extrapolated to others endemic areas with similar socioeconomic, cultural and health system problems, or rural and poor areas with lacking welfare state programmes. This critical research shows the urgency of creating and encouraging mechanisms to fight corruption; generating ruptures against the growing dependence on individualism, utilitarianism, and economism in health; and denature the fact that pregnant women must live exposed to MiP, since their PSDH are susceptible to transformation through efficient and equitable public policies.

## Conclusion

Most of the initiatives for MiP control are concentrated at individual level, PSDH identified in this research show the need to broaden the field of action of malaria programmes, increase health resources, and improve public health programmes and ANC. It is also necessary to impact the reciprocal relations of MiP with the economic and cultural dimension; although these aspects are increasingly diminished with the predominance and naturalization of neoliberal logics in health characterized by a growing dependence on individualism, utilitarianism and economism.

## Data Availability

All relevant data supporting the conclusions of this article are included within the article. Any additional information is available from the corresponding author upon reasonable request.

## Abbreviations

MiP: Malaria in pregnancy
PSDH: Processes of social determination of health
ANC: Antenatal care
QHR: Qualitative Health Research
HWs: Healthcare workers
HPE: Health promoter entity
HSPI: Health Services Provider Institutions
